# CuO@ZnO Nanocomposites with Improved Redox Behavior for High-Performance Supercapacitors

**DOI:** 10.3390/ma19122460

**Published:** 2026-06-09

**Authors:** Manesh A. Yewale, Santosh V. Mohite, Siham El Otmani, Annu, Dong Kil Shin

**Affiliations:** 1 School of Mechanical Engineering, Yeungnam University, Gyeongsan 38541, Republic of Korea; maneshphd@yu.ac.kr (M.A.Y.); sihamelotmani26@gmail.com (S.E.O.); drannu@yu.ac.kr (A.); 2 Department of Energy Material Science, Konkuk University, Chungju 27478, Republic of Korea; santoshmohite2989@gmail.com

**Keywords:** CuO@ZnO nanocomposite, transition metal oxides, energy storage devices, electrochemical kinetics

## Abstract

In this work, we employed an easy hydrothermal method to prepare CuO and ZnO, as well as the prepared composite nanostructured electrodes of CuO@ZnO for supercapacitor applications. The systematic electrochemical performance evaluation of the prepared materials was conducted by cyclic voltammetry (CV), galvanostatic charge–discharge (GCD), and electrochemical impedance spectroscopy (EIS). CuO@ZnO nanocomposite reflected the best charge storing behavior with a specific capacitance of 513 F/g, followed by pristine CuO (190 F/g) and ZnO (416 F/g). The composite also demonstrated 25.67 Wh/kg and 400 W/kg for energy density and power density, respectively, suggesting improved electrochemical performance. Besides, the areal and volumetric capacitances were 0.77 F/cm^2^ and 4.81 F/cm^3^, respectively, supported by the structural integrity and enhancement in electroactive materials utilization of the electrode material. Kinetic analysis showed that b values of the samples had mixed capacitive/diffusion-controlled charge storage, while higher diffusion coefficients and standard rate constants were apparent for ion transport or redox kinetics. EIS results showed a 2.14 Ω solution resistance, indicative of a decreased electrical resistivity. An asymmetric supercapacitor device fabricated by CuO@ZnO as the positive electrode and activated carbon (AC) as the negative electrode provided the specific capacitance of 48.57 F/g, energy density of 15.17 Wh/kg, and power density of 535 W/kg. After 10,000 cycles, the capacitance of the device was 76%, indicating good long-term stability.

## 1. Introduction

Currently, the surge of portable electronics, hybrid electric vehicles, and grid energy storage has a high demand for effective and renewable electrochemical storage systems. Supercapacitors have received considerable attention due to their high power density, quick charge–discharge property, and excellent cycling stability among all kinds of commercially available energy storage technologies [1,2,3,4]. However, their low energy density compared to traditional batteries continues to be a major challenge, thus driving the pursuit of novel electrode materials with enhanced electrochemical properties. Transition metal oxides (TMOs) with multiple oxidation states and rich redox chemistry have been studied as potential supercapacitor electrode materials for a long time [5,6,7]. Among them, copper oxide, a semiconductor characterized by a narrow bandgap, has shown high theoretical capacitance due to reversible Faradaic reactions. Nonetheless, low intrinsic electrical conductivity and structural damage during repeated charge–discharge cycling [8,9,10,11]. impair its actual electrochemical performance. ZnO, an n-type semiconductor with high electron mobility, good chemical stability, and environmental compatibility; however, its lower redox activity limits the capacitance contribution [12,13,14,15,16,17].

To tackle these limitations, numerous investigations on binary and hybrid metal oxide nanostructures for use in supercapacitors have been performed. CuO/ZnO heterostructures have recently been found to be an intriguing candidate as electrode materials because of the electrochemistry caused by the combination of the two oxides. Formation of CuO/ZnO heterointerfaces can facilitate electron transport, enhance charge separation, and functionally stabilize the structure during electrochemical cycling [18,19,20,21]. ZnO functions as a conductive and mechanically stable matrix design that relieves volume expansion of CuO, while the fast redox reactions with high reversibility contribute dominant pseudocapacitive performance. Accordingly, CuO/ZnO composites usually show better electrochemical kinetics and higher capacitance than pure materials. Although many CuO/ZnO composite electrodes have been reported, important limitations remain in previously published studies, such as intricate multistep synthesis strategies, insufficient interfacial coupling between CuO and ZnO phases, agglomerated morphologies with shorter ion diffusion pathways, and poor cycling stability at high current densities. Additionally, in various reports, the enhancement of electrochemical performance is often explained by a simple coexistence of CuO and ZnO, without an explicit correlation of morphological control and heterointerface engineering with charge storage behaviors. So, a simple and low-cost connected synthesis strategy for the successful formation of well-integrated CuO/ZnO heterostructures with increased electroactive surface area, high ion/electron transport efficiency, and improved structural stability still needs to be developed. Of the different fabrication methods, the hydrothermal one has garnered significant attention due to its simplicity, low cost, scalability, and capability to control the morphological size and shapes of particles with precision [22,23,24,25,26]. More importantly, hydrothermal growth enables close nanoscale interfacial contact between CuO and ZnO that is critical for facilitating charge-transfer kinetics on the electrode surface as well as optimizing synergistic electrochemical interactions. The rational morphology engineering from hydrothermal synthesis can also produce porous and continuous nanostructures that promote electrolyte infiltration and shorten the ions’ diffusion distance.

In this work, CuO, ZnO, and CuO@ZnO nanocomposite electrodes with varying ratios of the components were synthesized using a facile hydrothermal synthesis route to explore how heterointerface formation and morphology optimization affect supercapacitor performance. The novelty of the present work lies not only in the preparation of the CuO/ZnO composite but also in taking a step forward towards designing a highly interconnected heterostructure with better interfacial interaction and, consequently, enhanced electrochemical accessibility using an easily accessible approach. They were systematically characterized regarding structural, morphological, and compositional properties as well as investigated by electrochemical methods such as cyclic voltammetry (CV), galvanostatic charge–discharge (GCD), and electrochemical impedance spectroscopy (EIS). Experimental results reveal that the synergistic coupling between CuO and ZnO leads to dramatically improved charge-transfer properties, capacitance behavior, and cycling stability, suggesting that this rationally designed CuO-ZnO composite may be a powerful electrode material for supercapacitors.

## 2. Experimental

### 2.1. Synthesis of CuO Nanostructures

The copper oxide (CuO) nanostructures were synthesized via a controlled hydrothermal procedure. Typical of our procedures, the precursor reagents consisted of 0.1 M copper(II) nitrate trihydrate (Cu(NO_3_)_2_ 3H_2_O), 0.8 M urea (CO(NH_2_)_2_), and 0.4 M ammonium fluoride (NH_4_F). These essential components were fully dissolved in deionized (DI) water under continuous magnetic stirring, resulting in a transparent and homogenous solution that was uniform. The appropriately prepared solution was subsequently placed in a Teflon-lined stainless-steel autoclave, which was then sealed tightly to eliminate contamination. The autoclave was maintained at 140 °C for 7 h to promote crystalline growth via hydrothermal synthesis. After finishing this thermal process, the autoclave was allowed to cool naturally down to ambient temperature. The resulting precipitate was separated by centrifugation and thoroughly washed several times with DI water and ethanol to remove excess unreacted material, and finally dried at 60 °C for 12 h. The completely dried precursor was then heated at 400 °C for 4 h in a muffle furnace at a controlled heating rate to obtain phase-pure CuO.

### 2.2. Synthesis of ZnO Nanostructures

A similar hydrothermal process was used to fabricate ZnO nanostructures. In short, 0.1 M zinc nitrate hexahydrate (Zn(NO_3_)_2_ 6H_2_O), 0.8 M urea, and 0.4 M ammonium fluoride were mixed in DI water under constant stirring to produce a clear precursor solution that was homogeneously mixed throughout the solution volume. The obtained solution was subsequently transferred to a Teflon-lined autoclave and treated hydrothermally at 140 °C for 7 h. After this time, the autoclave was allowed to cool naturally, and the precipitate formed was separated by centrifugation. The precipitate was subsequently washed several times with DI water and ethanol for high purity. Lastly, the cleaned product was dried at 60 °C for 12 h. Finally, the powder was calcined at 400 °C for 4 h to afford well-crystallized ZnO nanostructures with a refined structure.

### 2.3. Synthesis of CuO@ZnO Composite

A heterostructured CuO@ZnO composite was prepared via a two-step hydrothermal method. The as-synthesized 20 mg CuO powder was first dispersed in DI water by ultrasonication to obtain a stable, homogeneous suspension. In a different procedure, an aqueous solution including 0.1 M zinc nitrate hexahydrate, 0.8 M urea, and 0.4 M ammonium fluoride was accurately prepared. Subsequently, this zinc precursor solution was slowly added to the CuO suspension while stirring continuously. The obtained mixture was then placed in a Teflon-lined autoclave and heated at 140 °C for 7 h for the in situ growth of ZnO onto CuO nanoparticles. After this thermal treatment, the composite product was collected by centrifugation, cleaned several times with DI water and ethanol, and then dried at a constant temperature of 60 °C for 12 h. Finally, the dried powder was calcined at 400 °C for 4 h to get the final CuO@ZnO heterostructured composite with highly crystalline and enhanced interfacial contact. The CuO@ZnO composite consisted of CuO and ZnO in a mass ratio of 1:3.55.

## 3. Results and Discussion

### X-Ray Diffraction (XRD) Structural Analysis

**Figure 1:**
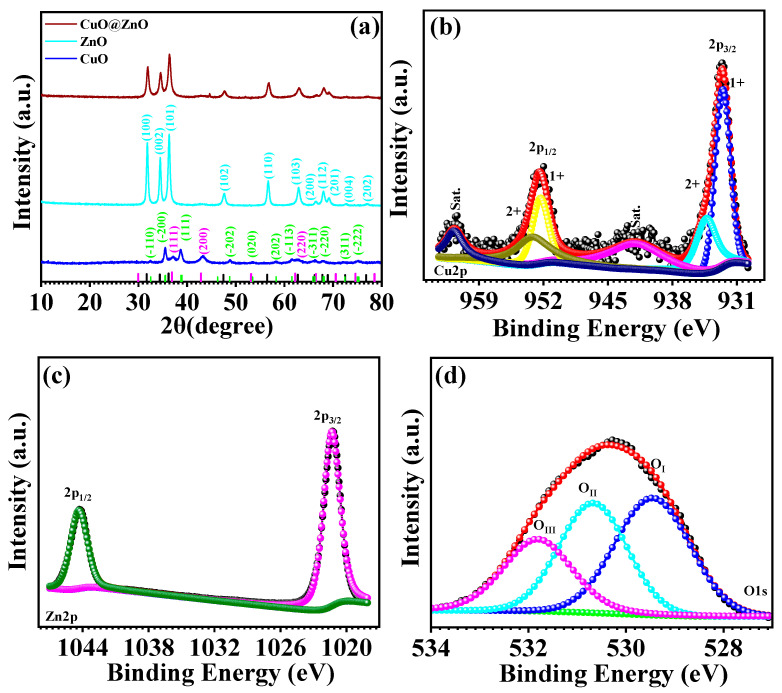
XRD spectra of CuO, ZnO, and CuO@ZnO composite (**a**), XPS spectra Cu 2p (**b**), Zn 2p, (**c**), and O 1s (**d**) of CuO@ZnO composite.

Phase purity, crystallographic structure, and successful composite formation were carefully analyzed by X-ray diffraction analysis, as shown in Figure 1a. Well-defined peaks seen in the diffraction patterns for pristine CuO, ZnO, and CuO@ZnO nanocomposite clearly show that all the synthesized materials under study are crystalline in nature. The diffraction peaks specific to the CuO sample at 2θ of 32.6°, 35.6°, 38.7°, 48.6°, 53.2°, 58.4°, 61.5°, and sometimes also for which different phases are observed, indicating various crystalline ionic structures with respect to the cubic or tetragonal symmetry. The peaks are indexed to the particular crystal planes like (−110), (−200), (111), (−202), (020), (202), (−113), (−311), (−220), (311), and (−222), confirming phase pure CuO formation. Additionally, the minor peaks detected at 2θ of 37.0°, 42.6°, and 62.4° can be attributed to Cu_2_O (JCPDS no. 00-034-1354), which is from the reflections of the planes (111), (200), and (220), respectively. Finally, the presence of Cu_2_O indicates that cupric (Cu^2+^) ions are at least partially reduced throughout hydrothermal synthesis, which has been noted frequently within controlled synthetic parameters. The observed peaks at 2θ of 31.77°, 34.32°, 36.29°, 47.57°, 56.67°, 62.94°, 66.35°, 68.04° 69.21°, 72.63°, and 76.89° are well indexed to the hexagonal wurtzite structure of ZnO according to JCPDS standard data (10790207) showing clear results in the diffraction pattern for as synthesized ZnO samples The lattice planes will be (100), (002),(101), (102), (110), (103), (200), (112), (201), (004), and (202) their diffracted angles such as they. Moreover, the intensity also correlates to high crystallinity and phase purity of ZnO, as in peak positions, no secondary phases are observed. As shown in Figure 1a, the diffraction peaks of CuO and ZnO phases are observed in the XRD pattern of CuO@ZnO composite, which demonstrates that the composite was formed without changing the crystal structures of the single constituents.

The absence of further peaks corresponding to impurities indicates high chemical compatibility of both components. Compared to ZnO, the characteristic peaks of CuO can also be observed in the diffraction pattern, confirming that both phases are well preserved on a composite structure. The slight change in peak positions and relative intensity of peaks in the composite compared to pristine materials can be ascribed to lattice strain and the strong interfacial interaction between CuO and ZnO. This construction provides an integral interfacial effect and consequently promotes electron transport across the heterojunction, critical to the material performance. Furthermore, the lack of peak broadening or evidence suggests that the hydrothermal route used permits controlled crystal growth, without compromising the crystallinity of the building blocks. The comparatively narrow peaks obtained from all the samples denote the formation of nanocrystalline with high crystallinity, which is most advantageous for electrochemical applications. Electrochemically, the existence of CuO as well as trace amounts of Cu_2_O, in parallel to ZnO within the composite, is beneficial. The pseudocapacitive behavior of CuO is attributed to the reversible redox couples of Cu^2+^/Cu^3+^, and ZnO strongly contributes to enhancing electron mobility and maintaining structural stability during charge-discharge processes. The presence of Cu_2_O could also further enhance additional redox activity and increase electrical conductivity, which is attributed to a mixed valence state-Cu(I)/Cu(II). Thus, the combined effect of CuO and ZnO phases would be expected to greatly improve the charge storage properties of the composite material. XRD spectra demonstrate the successful synthesis of a crystalline CuO@ZnO nanocomposite with a clearly defined heterostructure, which will undoubtedly aid in improving electrochemical properties due to its higher redox activity and favorable electronic transport pathways.

XPS studies of the CuO@ZnO composite reveal the surface chemical states and electronic environments of their respective elements in Figure S1 and Figure 1b–d. The survey-level binding energies (Cu 2p at 935.72 eV, Zn 2p at 1020 to 1044 eV, and O 1s at 531.69 eV) not only justified that both Cu and Zn were successfully doped into the multilayer matrix, but also ruled out other elemental impurities obtained from sources. The dynamic interaction between CuO and ZnO phases may introduce electronic coupling at the interface, which is advantageous for charge redistribution behaviors in terms of heterostructures. As shown in Figure 1b, the high-resolution Cu2p spectrum indicates several oxidation states of copper in the composite. In particular, the Cu2p_3/2_ peak at 932.97 eV contains a component centered at 932.47 eV, characteristic of Cu^+^ species, and the higher-binding-energy peak corresponds to Cu^2+^ (typically at 934.26 eV).

**Figure 2:**
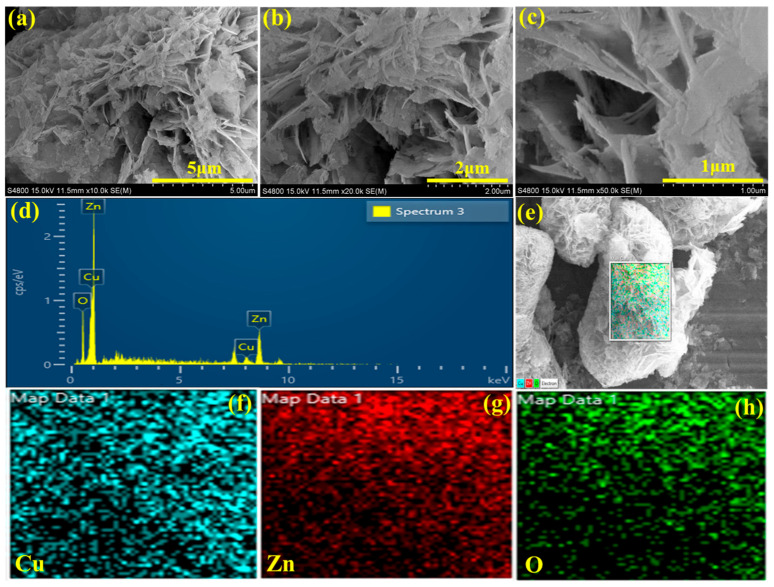
FESEM micrograph (**a**–**c**) of CuO@ZnO nanocomposite at different magnifications, EDS spectra of CuO@ZnO nanocomposite (**d**), elemental mapping of CuO@ZnO nanocomposite (**e**), elemental mapping of Cu (**f**), Zn (**g**), and O (**h**) of CuO@ZnO nanocomposite.

Simultaneously, the presence of Cu^+^ and Cu^2+^ refers to partial reduction or mixed valence states, which are typically linked with defect-rich structures and electrochemical charge transfer across the interspace. The notable intensity of the satellite peaks at 941.87 eV and 961.67 eV further supports the formation of Cu^2+^, as these shake-up satellites represent the Cu^2+^ electronic structure. Cu 2p_1/2_ region ranging from 952.29 eV, 952.64 eV, and 953.43 eV confirms the presence of two oxidation states of copper [27,28,29]. This mixed-valence characteristic is advantageous for their catalytic and electronic uses, where redox cycling is combined with enhanced charge-carrier dynamics. The Zn 2p spectrum is shown in Figure 1c. Two prominent peaks of Zn 2p_3/2_ at 1021.19 eV and at 1044.33 eV for Zn 2p_1/2_ can be seen. The energy difference between these two peaks (23.14 eV) agrees with the spin-orbit splitting of Zn^2+^ known from ZnO and indicates a stable oxidation state of Zn in the composite [30]. The lack of extra satellite features or peak asymmetry indicates that zinc is mainly present in a single chemical environment without considerable distortion due to defects [31,32,33]. This shows that the ZnO lattice retains its structure after being coupled with CuO and forms a distinct heterojunction. The O 1s spectra shown in Figure 1d, the peak could be divided into three parts, which are related to three types of O species present within the CuO@ZnO composite structure corresponding to different oxygen environments. There was a low binding energy peak corresponding to 529.47 eV (O_I_), which can be associated with the lattice oxygen (O^2-^) attached to metal ions present in the crystalline structure of CuO and ZnO. The peak O_II_ at 530.69 eV corresponds to oxygen vacancies or defect-related species, which serve a key function in tuning electronic structure and increasing surface activity. The peak with higher binding energy located at 531.81 eV (O_III_) is widely assigned to the presence of chemisorbed oxygen species on the surface, e.g., hydroxyl groups or adsorbed water molecules. The comparatively strong response of O_II_ and O_III_ indicates a high density of surface defects and active sites, which can greatly enhance the catalytic efficiency and adsorption behaviors [25,34,35]. XPS results support a CuO@ZnO composite with mixed copper valence states, stable Zn^2+^ species, and abundant oxygen defects. Such properties increase interfacial charge transfer and surface activity, both of which are suitable for energy storage. The surface morphology of the synthesized CuO, ZnO, and CuO@ZnO samples was systematically investigated by field-emission scanning electron microscopy (FESEM), as displayed in Figure S2a–f and Figure 2. The microstructural attributes of CuO and ZnO exhibited pronounced microstructural differences, which play a crucial part in differentiating their electrochemical activity.

The FESEM images for CuO (Figure S2a–c) reveal randomly shaped agglomerated nanoparticle structures with a rough and highly porous surface. The CuO sample presents a non-homogeneous size distribution with different particle sizes at lower magnification (Figure S2a), which reflects heterogeneous grain clustering. With higher magnification (Figure S2b,c), they seem to consist of interconnected nanoparticles that aggregate on each other, forming a less packed network. The reason for this agglomeration is that CuO nanoparticles have high surface energy in the hydrothermal growth process. This allows for a moderate surface area with the specific structural configuration, yet aggregation to excessive levels can lead to a dramatic decrease in accessible electroactive sites, as well as challenges associated with effective electrolyte diffusion. In comparison, the ZnO sample (Figure S2d–f) shows a distinct hierarchical morphology, comprising nanosheets or plate-like structures that are stacked in layers. Low-magnification microscopy images show that these form complicated flower-like assemblies (Figure S2d), and higher-magnification imaging clearly shows the presence of thin, extended nanosheets growing from nucleation sites at ambient conditions (Figure S2e,f). This organized morphology allows a higher surface-to-volume ratio, which promotes better pathways for ion transport. Also, the ZnO nanosheets with high structural integrity and uniformity lead to improved mechanical stability owing to the nature of using electrochemical cycling. The observed morphological differences highlight the respective benefits of these two materials, with CuO giving rise to redoxactive sites but suffering from aggregation tendencies, while ZnO serves as a stable, high-surface-area backbone effective for electrochemical processes. Such a contrast is of great significance toward the formation of CuO@ZnO composites, where ZnO acts as an effective supporting matrix to promote CuO dispersion and reduce its agglomeration tendency for overall improved electrochemical performance. Energy-dispersive spectroscopy (EDS) analysis was employed to confirm the pristine samples’ elemental composition, as shown in Figure S2g,h. 

In the case of the CuO sample (Figure S2g), several peaks corresponding to copper (Cu) and oxygen (O) are also detected, indicating successful formation of copper oxide with no detectable impurities. The ZnO sample (Figure S2h) shows only characteristic peaks of zinc (Zn) and oxygen, confirming the high purity of the synthesized zinc oxide. The notably absent contaminant elemental peaks across the individual spectra are clear indicators that the hydrothermally synthesized method is successful at producing chemically pure materials. Figure 2a–c show the FESEM images of the synthesized CuO@ZnO nanocomposite at different magnifications. The micrographs show a three-dimensional porous structure consisting of connected hexagonal ZnO nanosheets decorated with CuO nanoparticles. The morphology of the ZnO sheets is layered and wrinkled, resulting in a large number of open channels and pores that can assist electrolyte penetration and ion transport during charge–discharge processes. At higher magnifications (Figure 2b,c), it is clear that the two materials have not significantly agglomerated, with CuO nanoparticles uniformly anchored to the surface of the ZnO nanosheets. The ZnO nanosheets were determined to have a thickness of 46.27 nm, which explains the rugged morphology and high surface area-to-volume ratio inherent in ultrathin layered structures. The FESEM images revealed that, in addition to spatially homogeneous ZnO, uniform CuO nanoparticles with a particle size of 35 nm were also observed. The nanoscale size of the CuO particles affords ample electroactive sites, and their close contact with ZnO nanosheets ensures facile charge transfer across the heterostructure. The EDS spectrum of CuO@ZnO nanocomposite is presented in Figure 2d with its elemental composition. Analysis of the spectrum clearly reveals that there are no detectable impurity peaks and that only Cu, Zn, and O elements were seen, confirming the high purity of the synthesized material. The strong peaks assigned to Cu and Zn confirm the incorporation of both metal oxides into the composite framework successfully. The peak of oxygen also confirms the formation of oxide phases.

**Figure 3:**
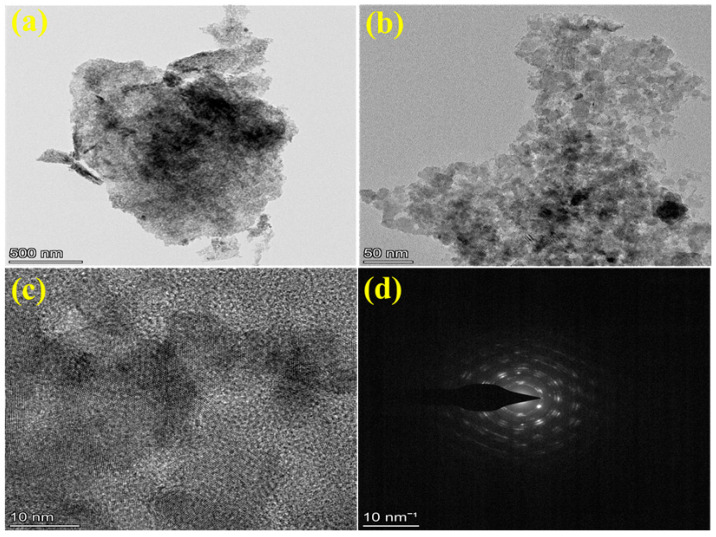
TEM image (**a**) low-magnification, (**b**,**c**) high-magnification, (**d**) SAED pattern of CuO@ZnO composite.

Elemental mapping analysis was performed to further explore the spatial distribution of elements in the composite (Figure 2e–h). The mapping images convincingly show the even distribution of Cu (Figure 2f), Zn (Figure 2g), and O (Figure 2h) in the selected area of the nanocomposite. This uniform distribution of the elements shows that a well-integrated heterostructure is formed instead of just a physical mixture. Such homogeneity is critical to provide efficient interfacial contact between CuO and ZnO to enable charge-transfer kinetics. The close contact between CuO and ZnO phases, presented in the homogeneous elemental mapping distribution, is expected to promote strong interfacial coupling. This interaction would improve charge transit through the heterojunction while facilitating combined redox activity. The homogeneous contribution allows full usage of the benefits that both materials can achieve across the whole electrode material. Moreover, this nanosheet-based morphology and uniform elemental distribution promote the formation of active sites that are favorable for electrochemical reactions. In addition to enhancing the specific capacitance of this configuration, the rate capability is also improved by fast ion adsorption/desorption and electron transfer. The FESEM analysis indicates the formation of a porous stack nanosheet structure, whereas EDS and elemental mapping support uniform composition distribution for the successful integration of Cu, Zn, and O elements. Morphological and structural properties of the synthesized CuO@ZnO composite were investigated by transmission electron microscope (TEM) along with selected area electron diffraction (SAED), as shown in Figure 3a–d). Source: The low-magnification TEM image in Figure 3a shows the formation of a highly agglomerated, cloud-like structure composed of connected nanostructures. The assembly of high-surface-energy nanoscale constituents into dense clusters also promotes better electrical contact among the various particles, which is critical to charge transport at supercapacitor electrodes. At higher magnification (Figure 3b), it is shown that the composite nanomaterials are uniformly dispersed quasi-spherical nanoparticles in the nanometer range. These nanoparticles seem to develop an open structure, creating interparticle voids that can serve as ion diffusion pathways. This porous morphology is advantageous for electrochemical applications, facilitating electrolyte access and reducing ion transport delay to boost capacitive performance.

To further understand the microstructural characteristics of the CuO@ZnO nanocomposite, TEM and SAED analyses were performed, shown in Figure 3a–d. As seen in the low-magnification transmission electron microscopy (TEM) image (Figure 3a), a very porous, loosely packed morphology of interlinked ultrathin nanosheets can be observed. This type of structure is advantageous for electrochemical applications because it provides a large interface area while also allowing for fast transport of ions through the active material. The TEM image at higher magnification (Figure 3b) shows that many nanosized CuO particles are dispersed uniformly on the ZnO nanosheet matrix. The CuO nanoparticles on them, which have higher electron density, are in darker contrast, and no serious agglomeration takes place, but they disperse well. The uniform distribution of CuO nanoparticles throughout the ZnO nanosheets enhances the electroactive surface area and ensures rapid utilization of the overall active material during charge-storage processes. The TEM image shows bright lattice fringes, which confirm the high crystallinity of the composite (Figure 3c). The interplanar spacings observed correspond to characteristic planes of ZnO and CuO crystals, indicating the coexistence of both phases in a heterostructure. These features of intimate interfacial contact between CuO and ZnO provide fast electron-transport pathways and effective charge transfer across the heterojunction, both of which are essential for promoting electrochemical performance. The SAED pattern in Figure 3d provides additional structural confirmation. The multiple concentric diffraction rings with several bright diffraction spots characteristic of a polycrystalline nature of the nanocomposite can be observed on its electron-diffraction image. Both sets of diffraction rings can be indexed to the crystallographic planes that correspond with CuO or ZnO, confirming a crystalline CuO@ZnO heterostructure is formed. No further diffraction features indicate phase purity of the synthesized material. The TEM analysis shows that this unique microstructure has significant potential for energy-storage applications. These ultrathin ZnO nanosheets offer a high accessible surface area and short ion-diffusion distances, while the uniformly distributed CuO nanoparticles add more than just faradaic redox-active sites. Additionally, the heterointerface of CuO/ZnO improves charge-transfer kinetics and hinders particle aggregation during the cycling test. The porosity in the architecture helps with the volume changes that accompany redox reactions, therefore enhancing structural stability as well.

## 4. Electrochemical Analysis

**Figure 4:**
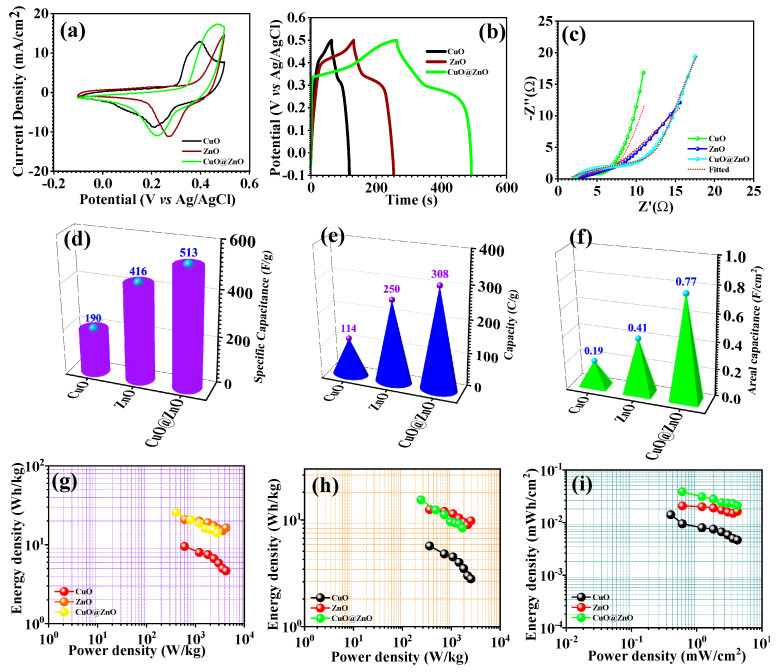
Comparative CV profile (**a**), GCD profile, (**b**) EIS spectra (**c**), comparative specific capacitance (**d**), specific capacity (**e**), areal capacitance (**f**) at 2mA/cm^2^, Ragone plot corresponding to C_s_ (**g**), C (**h**), and C_a_ (**i**) of CuO, ZnO, and CuO@ZnO electrode.

To achieve a better understanding of the complex electrochemical behavior of CuO, ZnO, and CuO@ZnO electrodes, we systematically investigated using cyclic voltammetry (CV), galvanostatic charge-discharge (GCD), and electrochemical impedance spectroscopy (EIS), as shown in Figure 4a–c. Furthermore, the electrochemical impedance spectroscopy spectra, as shown in Figure 4c, provide further elucidation of the inherent charge transfer properties of the materials. The Nyquist plots show a semicircular area in the high frequency region, followed by a straight line in the low frequency. From these, the solution resistance (R_s_) values are found to be 2.63 Ω for CuO, 2.49 Ω for ZnO, and remarkably low at 2.14 Ω for the CuO@ZnO composite. The corresponding decreased R_s_ in the composite reveals a more conductive pathway with lowered internal resistance, which should be ascribed to the enriched interfacial connectivity by forming a heterointerface between CuO and ZnO. 

Moreover, the slope in the low-frequency region of the composite is significantly steeper, indicating improved ion diffusion and capacitive behavior. Figure S3a–c shows the CV profile of CuO, ZnO, and CuO@ZnO at different scan rates. The comparative cyclic voltammetry (CV) profiles shown in Figure 4a exhibit clearly distinguishable redox peaks for all three types of electrodes, lending further affirmation to their inherent pseudocapacitive properties. It is important to highlight that the CuO@ZnO composite exhibits a much larger integrated area underneath the CV curve than both pristine CuO and ZnO, indicating a better ability for charge storage. The distinctive redox peaks also suggest that the charge storage mechanism is mainly caused by the reversible faradaic reaction during the transformation of Cu^2+^ to Cu^3+^ ions, while ZnO serves a crucial role in enhancing charge transport and maintaining structural reliability. The galvanostatic charge–discharge profiles evaluating the samples, as illustrated in Figure 4b, also support the results collected from cyclic voltammetry and Figure S3d–f GCD profile of CuO, ZnO, and CuO@ZnO at different current densities. The non-linear charge–discharge curves exhibited by each of these electrodes signify battery-type behavior. Out of these electrodes, the CuO@ZnO composite exhibits a significantly larger discharge duration, representing enhanced capacitance. The comparative C_s_, C and C_a_ at 2 mA/cm^2^ are shown in Figure 4d–f and different current densities in Figure S4a–d [36,37,38] obtained from Equation (S1a–d) (Supporting Information (SI)). The comparative C_v_ and corresponding Ragone plot are shown in Figure S4e,f. The specific capacitance (C_s_) values estimated based on the GCD measurements indicate 190 F/g for CuO, 416 F/g for ZnO, and a value of 513 F/g was also calculated from that series of data for the CuO@ZnO composite. This improvement in the electrochemical performance of the composite electrode can be attributed to a combined effect of CuO and ZnO, with CuO providing numerous redox-active sites and ZnO promoting electron transport and reducing structural degradation. The specific capacity values also increase in a similar pattern, reaching 114 C/g for CuO, 250 C/g for ZnO, and 308 C/g for CuO@ZnO. Such a significant enhancement reveals the advantages of both electric double-layer capacitance and the Faradaic mechanism in the composite electrode. In addition, the areal capacitance rises significantly from 0.19 F/cm^2^ to 0.77 F/cm^2^ for CuO and the composition of ZnO@CuO, respectively, as well as volumetric capacitance also improves four times between these two compounds (from 1.18 F/cm^3^ to 4.81 F/cm^3^), indicating charge storage performance at surface-based and bulk scales concurrently. Figure 4g–i shows the Ragone plots, which demonstrate the energy density versus power density for different types of electrode material obtained from Equations (S2) and (S3) (SI). The specific energy density of the CuO@ZnO composite reaches 25.67 Wh/kg, much higher than that of CuO (9.5 Wh/kg) and ZnO (20.83 Wh/kg). While the composite boasts a slightly lower power density (400 W/kg) compared to its constituents (600 W/kg), it still maintains respectable energy/power capabilities. Likewise, the areal and volumetric energy densities of this composite were significantly enhanced, reported at 0.04 mWh/cm^2^ and 0.2406 mWh/cm^3,^ respectively, which demonstrates its potential for compact energy storage devices.

**Figure 5:**
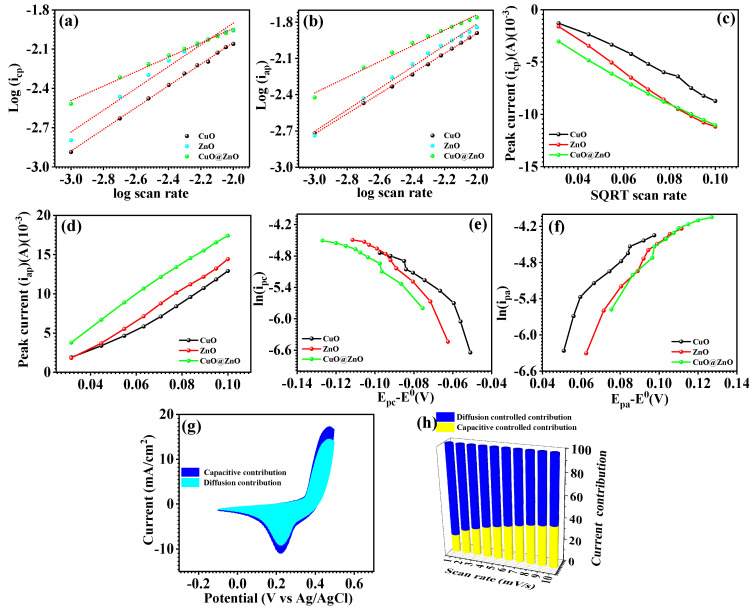
Comparative b value plot cathodic (**a**), anodic (**b**), diffusion coefficient plot cathodic (**c**), anodic (**d**), k^0^ and α value plot cathodic (**e**), anodic (**f**) of CuO, ZnO, and CuO@ZnO electrode, capacitive and diffusion current contribution at 10mV/s scan rate (**g**), and different scan rate (**h**) of CuO@ZnO electrode.

Cyclic voltammetry (CV) curves with varying scan rates were obtained to probe the charge-storage mechanism and electrochemical kinetics as well. Power-law relationship between the peak current (i) and scan rate (*v*) (Equation (S4)). The b-value sheds light on what charge-storage mechanism is controlling a reaction, with b = 0.5 corresponding to a diffusion-controlled process and b = 1.0 indicating a surface capacitive effect controlling the process [39,40,41,42,43]. The b-values calculated from the slopes of log(i) vs. log(v) plots were represented in Figure 5a,b as the anodic 0.83, 0.87, and 0.65 for CuO, ZnO, and CuO@ZnO, respectively, or cathodic 0.82, 0.83, and 0.55, respectively. All electrodes demonstrate both capacitive and diffusion-controlled contributions to charge storage, as indicated by these results. When compared to the CuO@ZnO composite, which has lower b-values (0.5), the higher b-values of ZnO favor a greater contribution from diffusion-controlled Faradaic reactions and thus a smaller capacitive contribution. Such behavior is favorable for pseudocapacitive energy storage, considering that the hierarchical porous structure of CuO@ZnO allows fast ion diffusion through the entire electrode while having a corresponding large surface area.

To quantify the ion transport properties, the determined D values obtained from Equation (S5) were used to evaluate diffusion coefficients and plotted in Figure 5c,d. The anodic diffusion coefficients increase from 8.92 × 10^−7^ cm^2^/S for CuO to 1.12 × 10^−7^ cm^2^/S for ZnO and then to 1.25 × 10^−7^ cm^2^/S for CuO@ZnO, respectively [42,43,44]. Since the composite shows a higher diffusion coefficient than CuO and ZnO, this can be explained by high ion mobility due to the porous interconnected network. The open channels allow the electrolyte to penetrate easily, thereby lowering the diffusion barrier and enhancing the utilization of electroactive sites. The cathodic diffusion coefficient of CuO@ZnO (5.8 × 10^−7^ cm^2^/S) is smaller than those values obtained for the pristine materials, but it also shows both lower analyzed layers, demonstrating to confirm that the comprehension investigated efficient ionic transport during the oxidation process and is directly related to higher charge-storage ability in the composite electrode significantly. For the kinetic evaluation of reversibility and electron-transfer kinetics for redox reactions, we determined each standard heterogeneous rate constant (k^0^) and charge-transfer coefficient (α) from the CV data using Equation (S6) and Figure 5e,f. The k^0^ values were close to those found for quasi-reversible electrochemical processes, being on the order of 10^−5^ to ×10^−1^ cm/S. More particularly, the anodic k_0_ are of 0.33 × 10^−5^ to 0.58 × 10^−5^ cm/S, while those for cathodic are from 0.37 × 10^−5^ cm/S to 1.02 × 10^−5^ cm/S [45,46,47]. Higher k^0^ values for CuO@ZnO compared with those of the single CuO and ZnO electrodes suggest faster interfacial electron-transfer kinetics. The improvement, ascribed to charge transport through the heterojunction between CuO and ZnO, is also reflected in the reduced charge-transfer resistance of the electrode/electrolyte interface. The results on charge-transfer coefficient (α) values provide further insight into reaction kinetics and the reversibility of redox processes [47,48,49,50]. Anodic α values between 0.76 and 0.96 vary, while the cathodic values range from 0.64 to 0.99. All values are within the physically reasonable range of 0–1; this indicates some degree of quasi-reversibility of electrochemical reactions. The lower α value of CuO@ZnO than pristine ZnO indicates much more even charge-transfer kinetics and higher redox reversibility. Its unique electrochemical property with superior ion diffusion, high electron mobility, and good reversibility of Faradaic reactions by the CuO@ZnO composite contributes to the approach. The capacitive behavior of CuO@ZnO stems overall from the combined effects of CuO and ZnO, hierarchical porous nanosheet morphology, increased electroactive surface area, enhanced accessibility of electrolyte to electrode material, improved ion diffusion, and increased kinetics for interfacial charge-transfer reactions. These characteristics all contribute to a higher specific capacitance, enhanced rate performance, increased energy density, and better cycling stability than the single electrodes of CuO and ZnO. Additionally, the contributions of capacitive and diffusion-controlled mechanisms were quantitatively evaluated using Dunn’s method as shown in Figure 5g,h, and Equation (S7) (SI). The contribution from surface-controlled (capacitance-derived) and diffusion-controlled processes is significant at a scan rate of 10 mV/s, as evidenced by the current response of the CuO@ZnO electrode. It is clear that while the capacitive contribution predominates at high scan rates, contributions from diffusion-controlled processes become more relevant at lower scan rates. Figure 5h directly shows the corresponding bar plot, with an increase in capacitive contribution at higher scan rates, indicating that this electrode could efficiently promote rapid surface redox and bulk diffusion processes. The CuO@ZnO composite electrode achieved exceptionally improved electrochemical properties due to finely balanced capacitive and diffusion-controlled mechanisms, accelerated ion diffusion ability, and rapid electron transfer kinetics.

### Asymmetric Supercapacitor (ASC)

**Figure 6:**
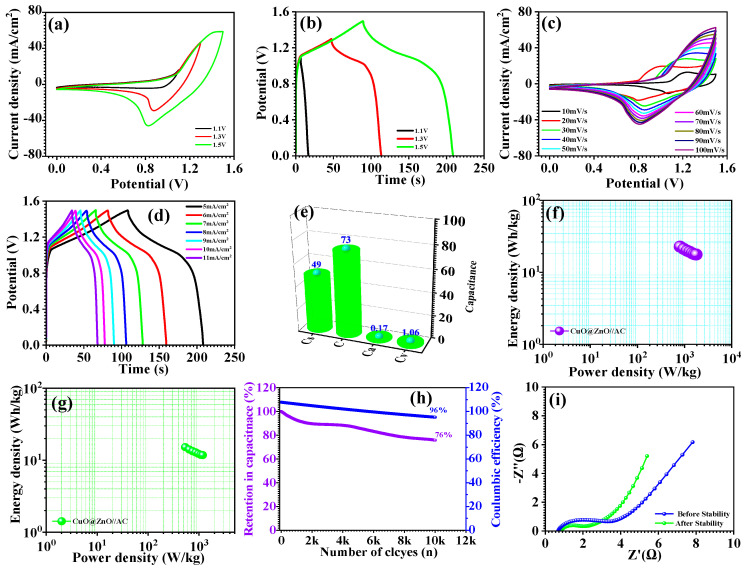
CV profile at different potential window (**a**), GCD profile at different potential window (**b**), CV profile at different scan rate (**c**), GCD profile at different current density (**d**), comparative capacitance C_s_, C, C_a_, and C_v_ (**e**), Ragone plot corresponding to C_s_ (**f**), C (**g**), cyclic stability and columbic efficiency (**h**) and EIS spectra before and after cyclic stability (**i**) of CuO@ZnO//AC ASC.

Figure 6 shows the electrochemical performance of CuO@ZnO//AC asymmetric supercapacitor (ASC) characterized by cyclic voltammetry (CV), galvanostatic charge-discharge (GCD), electrochemical impedance spectroscopy (EIS), and long-term cycling tests. The charge balance was calculated from Equation (S8), and the mass ratio of the positive to negative electrode was maintained at 1:2.04 with a total loading mass of the electrode of 7 mg. As seen in Figure 6a, the CV curves recorded between different potential windows appear to be quasi-rectangular with notable redox peaks within which the electric double-layer capacitance contribution from the activated carbon electrode cooperatively couples with the Faradaic pseudocapacitance of the CuO@ZnO electrode. The retention of the CV shape in the selected operating voltage range indicates satisfactory electrochemical reversibility and limited polarization. In addition, the CV profiles at different scan rates (Figure 6c) have nearly identical shapes, with only slight distortion at high scan rates due to kinetic limitations, indicating relatively fast ion diffusion and charge-transfer processes. The GCD curves (Figure 6b,d) reveal non-linear and close to symmetrical charge-discharge behavior characteristic of the pseudocapacitive nature of the device. The ASC provides a specific capacitance of 48.57 F/g (Figure 6e) and calculated according to the GCD results, a specific capacity of 72.86 C/g in this manner. 15.18. Wh/kg energy density is obtained at 535.71 W/kg power density. To assess the practical relevance of this result, Table 1 compares these values with recently reported copper-based supercapacitors, showing that the energy density results are in line with other transition metal oxide-based supercapacitors and demonstrate competitive electrochemical performance. Beyond gravimetric metrics, it demonstrates an areal capacitance of 0.17 F/cm^2^ with a maximum areal energy density of 0.053 mWh/cm^−2^ and power density of 1.875 mW/cm^2^, as well as 1.06 F/cm^3^ volumetric capacitance, 0.33 mWh/cm^3^ energy density, and 11.72 mW/cm^3^ power density.

The expected tradeoff between energy and power densities, where higher power output goes in bulk at the expense of some energy density, is shown in Ragone plots (Figure 6f,g and Figure S5). The underlying reason for this behavior is due to the combination of charge-storage mechanisms of the battery-type CuO@ZnO electrode and the capacitive activated carbon electrode. The cycling performance was also measured over 10,000 charge-discharge cycles, which indicates long-term device stability (Figure 6h) and reveals that the device retains 76% of its initial capacitance. While this retention is evidence for electrochemical activity, it may be considered moderate. This decay in capacitance originates from structural changes in the active material, the gradual loss of electrochemically accessible sites, and interfacial degradation that occurs throughout long-term cycling. This gives you a corresponding coulombic efficiency of 96%. The EIS spectra (Figure 6i) yield a clear understanding of the interfacial electrochemical properties of the CuO@ZnO//AC ASC before and after cycling. The resistance of the electrolyte (R_s_) rises only a little bit from 0.65 Ω to 0.86Ω, which confirms that during long-term cycling, mainly only minor changes in the resistances of the electrodes/electrolyte and electrode/current-collector contacts take place. 

Overall, the Nyquist plots indicate that the charge-transfer resistance (R_ct_) decreases from 2.81 Ω to 1.08 Ω after cycling. Although a smaller Rct is always beneficial for charge transfer processes, this result should be read carefully. The decrease can be attributed to a combination of electrode activation, improved wetting of the porous structure by electrolyte, and structural rearrangements from cycling that couple greater accessibility to electroactive sites with more rapid ion/electron transport. As such, the changes in impedance after cycling relate not only to improved intrinsic charge-transfer kinetics but also to improved interfacial electrochemical behavior. A smaller semicircle diameter in the high-frequency region confirms a decrease in interfacial resistance and an increase in electrode utilization after repeated cycling. The balanced combination of energy-storage and power-deliver capability of CuO@ZnO//AC ASC before 10,000 cycles, with moderate cycling stability, still requires optimizing the structure of electrodes and interfacial stability, dealing with submergence in a long-term operation.

## 5. Conclusions

In this work, electrode materials based on three different systems, namely CuO, ZnO, and a CuO@ZnO composite, were successfully synthesized using a hydrothermal method for supercapacitor applications. The formation of the heterostructured composite framework was confirmed through structural, morphological, and compositional characterizations. The enhanced electrochemical performance is attributed to the combined effects of the porous hierarchical morphology, uniform elemental distribution, and intimate interfacial contact between the two metal oxides. Electrochemical investigations indicate that the improved charge-storage performance of the CuO@ZnO composite arises from the synergistic contributions of both components. CuO provides redox-active sites for Faradaic charge storage, while ZnO contributes to structural stability and facilitates charge transport. Kinetic analyses revealed a mixed capacitive and diffusion-controlled charge-storage mechanism, highlighting the contributions of both surface and bulk electrochemical processes to energy storage. The fabricated CuO@ZnO//AC asymmetric supercapacitor exhibited moderate energy-storage performance, delivering an energy density of 15.18 Wh/kg at a power density of 535.71 W/kg. Although these values compare favorably with those reported for several transition-metal oxide-based supercapacitor systems, the device retained only 76% of its initial capacitance after 10,000 charge-discharge cycles, indicating that further refinements are required to improve long-term cycling stability. This study demonstrates that constructing a CuO-ZnO heterostructured composite is an effective strategy for enhancing electrochemical charge-storage performance. The findings provide valuable insights into the design of binary transition-metal oxide electrodes and may serve as a foundation for the future development of supercapacitor materials with improved durability and enhanced electrochemical performance.

## Figures and Tables

**Table 1:** Comparative study of an asymmetric supercapacitor.

Electrode	Method	Electrolyte	Energy Density (Wh/kg)	Power Density (W/kg)	Ref.
CuO@ZnO	Hydrothermal	2 M KOH	15.18	535	Present work
Cu-MOF/rGO nanoparticles	Ultrasonication	1 M Na_2_SO_4_	14.59	12,000	[51]
Graphene@Cu_2_O	Electrodeposition	6 M KOH	6.63	323	[52]
CuCo_2_O_4_	Electrodeposition	6 M KOH	5.98	4500	[53]
Cu_2_S	Sulfuration	2 M KOH	4.7	288	[54]
CuCo_2_O_4_	Solution combustion method	3 M KOH	3.05	22,110	[55]

## Data Availability

The original contributions presented in this study are included in the article/supplementary material. Further inquiries can be directed to the corresponding author.

## Supplementary Materials

The following supporting information can be downloaded at: https://www.mdpi.com/article/10.3390/ma19122460/s1. Figure S1: Survey XPS spectra of CuO@ZnO composite; Figure S2: FESEM micrograph of CuO (**a–c**), ZnO (**d–f**), EDS spectra of CuO (**g**), ZnO (**h**); Figure S3: CV profile of CuO (**a**), ZnO (**b**), CuO@ZnO (**c**), GCD profile of CuO (**d**), ZnO (**e**), CuO@ZnO (**f**); Figure S4: Comparative Cs (**a**), C (**b**), Ca (**c**), Cv (**d**) at different current density of CuO, ZnO And CuO@ZnO electrode, Cv at 2mA/cm2 and Ragone plot corresponding to Cv of CuO, ZnO And CuO@ZnO electrode; Figure S5: Ragone plot corresponding to Ca (**f**), and Cv (**g**) of CuO@ZnO//AC ASC.
