# Supplementary material for: CuO@ZnO Nanocomposites with Improved Redox Behavior for High-Performance Supercapacitors
Source: Materials (Basel). 2026 Jun 9;19(12):2460. doi: 10.3390/ma19122460 (PMC13301693; doi:10.3390/ma19122460)
Supplement: Supplementary file 1 [file materials-19-02460-s001.zip › materials-4297726-supplementary.pdf]

Supporting Information

CuO@ZnO Nanocomposites with Improved Redox Behavior for High-Performance Supercapacitors

Manesh A. Yewale[3*], S. V. Mohite[2], Siham El Otmani[3], Annu[3], D. K. Shin[3*]

[1]School of Mechanical Engineering, Yeungnam University, Gyeongsan 38541, Republic of Korea
[2]Department of Energy Material Science, Konkuk University, Chungju, 27478, Republic of Korea
[2]Department of Chemistry, Gachon University, Republic of Korea
Corresponding author: dkshin@yu.ac.kr

Chemical

All the reagents were used without additional purification purchased from daejung chemicals, south korea, Manganese nitrate ($Mn(NO_3)_2$, copper nitrate ($Cu(NO_3)_2$, urea (CH_4N_2O), ammonia fluoride (NH_4F), ethanol (C_2H_5OH), acetone (C_3H_6O). hydrochloric acid (HCl). Polyvinylidene fluoride (PVDF), Nmethyl-2-pyrrolidone (NMP) and potassium hydroxide (KOH) were bought from SigmaAldrich.

Material characterization

The physicochemical properties of samples were explored by applying several technologies including specific qualitative and quantitative methods for their analysis. XRD measurements with CuK_α radiation using a Panalytical diffractometer were performed to investigate structural phase evolution. The oxidation and stoichiometry of the materials was performed with K-alpha XPS technology from Thermo Fisher Scientific. Morphological analysis was performed using a FESEM (S-4800 HITACHI, Ltd., Japan), while sample quality was characterized using an HR-TEM (Tecnai F21, FEI Company). Preparation and characteristic of SAED pattern Lastly, the electrochemical properties were examined by a WonAtech ZIVE SP5 electrochemical workstation.

Electrode preparation and electrochemical measurement

Electrochemical experiments have been performed with different CuO, Mn_2O_3 and CuO@Mn_2O_3 composite electrodes in 2- and 3-electrode systems. A three-electrode system consisting of a working electrode based on CuO, Mn_2O_3 and CuO@Mn_2O_3 materials, platinum as the counter electrode and Ag/AgCl as the reference electrode. In this two-electrode set-up, the best electrode (found in the three electrode experiment) was used as a working electrode, while activated carbon (AC) acted as counter electrode. For all electrochemical experiments, a 2 M KOH solution was used as the electrolyte. The method of preparing the working and activated carbon electrodes was similar in all cases. The active ingredient CuO, Mn_2O_3 and CuO@Mn_2O_3 were mixed with PVDF and carbon black at a mass ratio of 8:1:1 in NMP as solvent. To constantly disperse the contents, one hour ultrasonicated solution was used. Nickel foam was ultrasonically cleaned in acetone, ethanol and distilled water for 30 minutes each prior to electrode drop casting. After that, it was dried at 60°C for 5 hours. The working electrode was obtained by evaporating the slurry on a 1×1 cm^2 Ni foam and drying it overnight at 60 ºC. This void was employed for subsequent electrochemical assays. and more details regarding electrochemical measurement, and the fabrication and testing of the asymmetric supercapacitor device are given in the Supporting Information

Equations

Specific capacitance (C_s) $= \frac{I \times \Delta t}{m \times \Delta V}$ -- (S1a)

Specific capacity (C) $= \frac{I \times \Delta t}{\Delta V}$-- (S1b)

Areal capacitance (C_a) $= \frac{I \times \Delta t}{A \times \Delta V}$-- (S1c)

Volumetric capacitance (C_v) $= \frac{I \times \Delta t}{V \times \Delta V}$-- (S1d)

Energy Density ($ED_{s,c,a,v}$)= $\frac{C_{s,c,a,v} \times V^2}{7.2}$-- (S2)

Power Density ($PD_{s,c,a,v}$)= $\frac{ED_{s,c,a,v} \times 3600}{\Delta t}$-- (S3)

$i_p = a v^b$ --(S4)

$\frac{i_p}{\sqrt{v}} = 2.69 \times A \times C \times \sqrt{D} \times \sqrt{n}$--- (S5)

$i_p = 0.227 ACFnk^0 \exp\left[-\alpha nF/RT(E_p - E^{\circ})\right]$ ---(S6)

$i_p(V) = k_c v + k_d \sqrt{v}$---(S7)

The mass balancing is needed to optimize the capacitive behaviors of hybrid devices. Mass balancing of material was carried out using the following equation.

$$\frac{m_+}{m_-} = \frac{C_- \times \Delta V_-}{C_+ \times \Delta V_+} \qquad (S8)$$

Where m $_{(+/-)}$ is the mass of active material, C $_{(+/-)}$ is the specific capacitance, and ΔV $_{(+/-)}$ is the potential window.

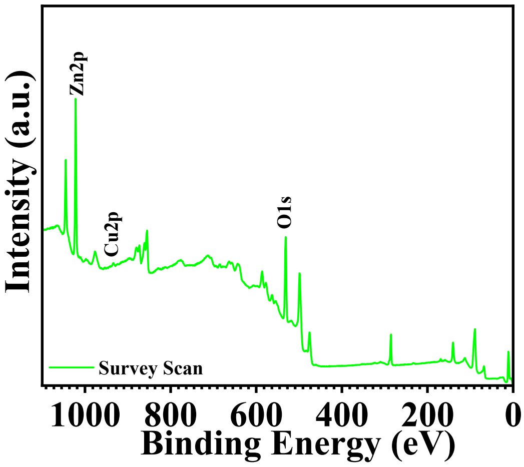

Figure S1: Survey XPS spectra of CuO@ZnO composite

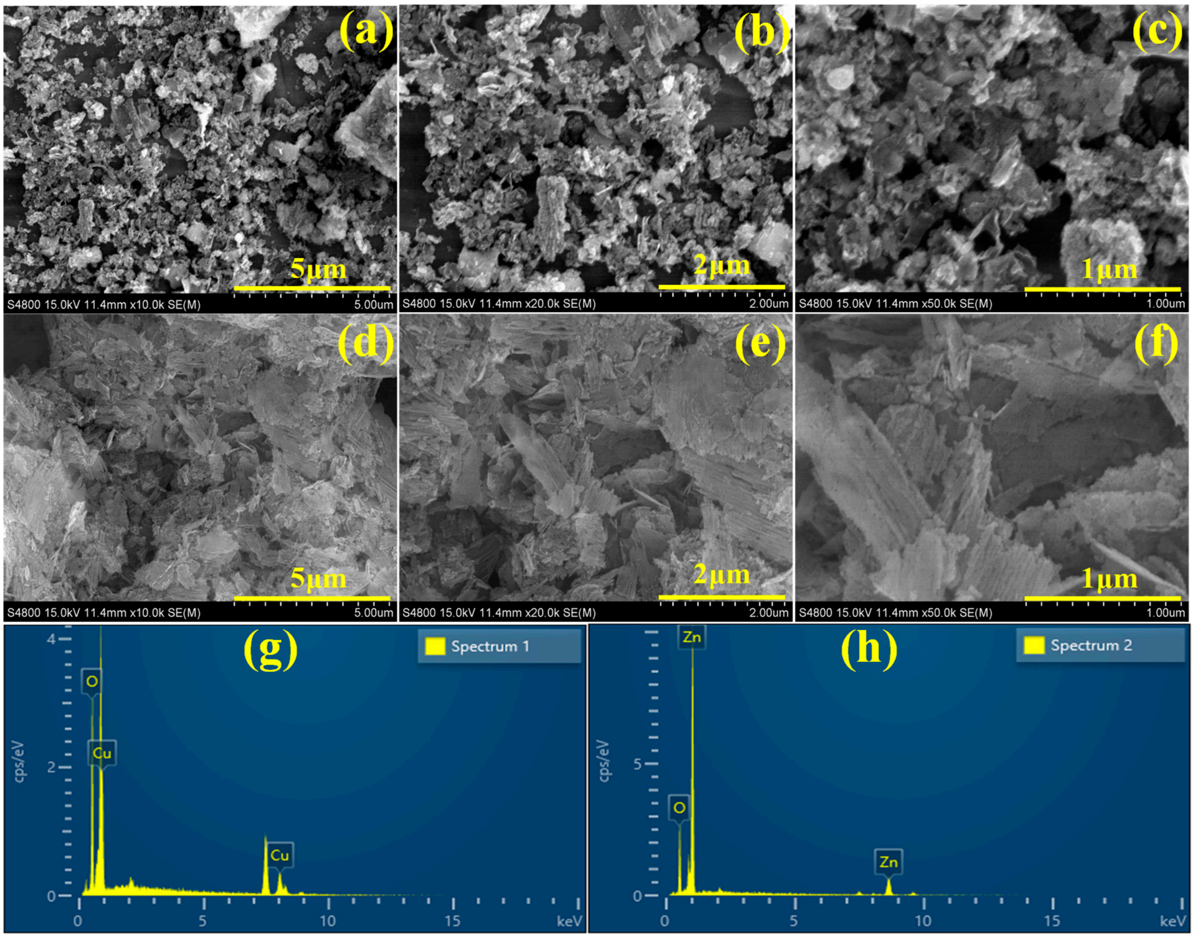

Figure S2: FESEM micrograph of CuO (a-c), ZnO (d-f), EDS spectra of CuO (g), ZnO (h)

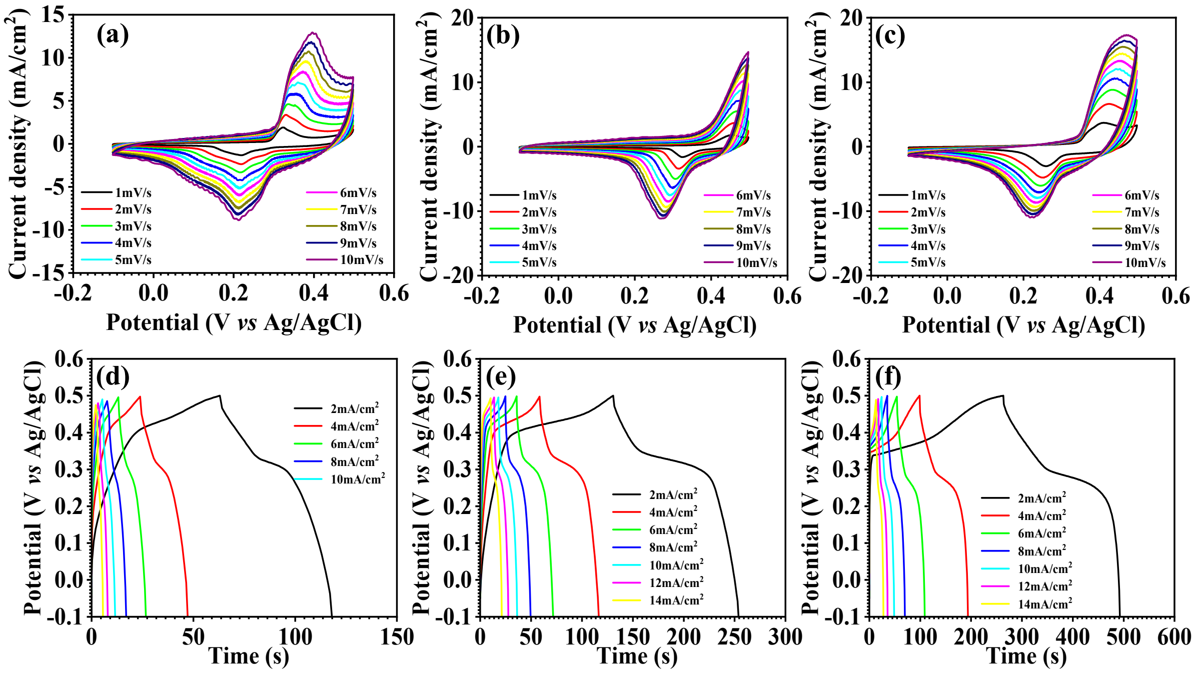

Figure S3: CV profile of CuO (a), ZnO (b), CuO@ZnO (c), GCD profile of CuO (d), ZnO (e), CuO@ZnO (f)

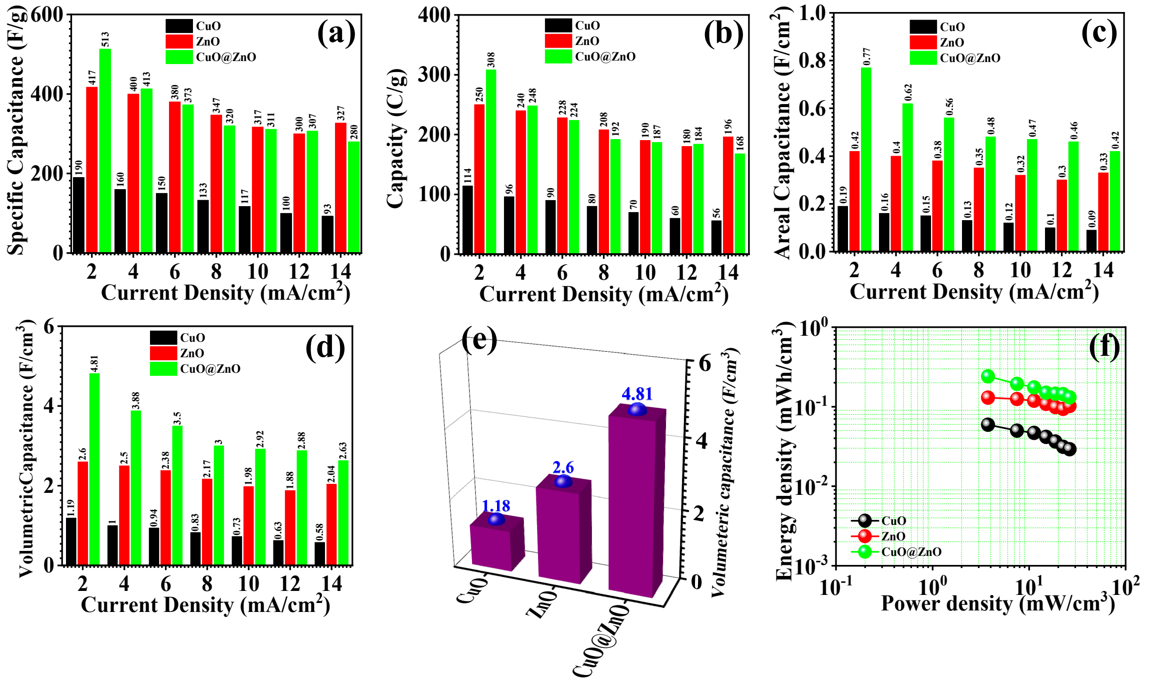

Figure S4: Comparative C_s (a), C (b), C_a (c), C_v (d) at different current density of CuO, ZnO And CuO@ZnO electrode, C_v at 2mA/cm^2 and Ragone plot corresponding to C_v of CuO, ZnO And CuO@ZnO electrode

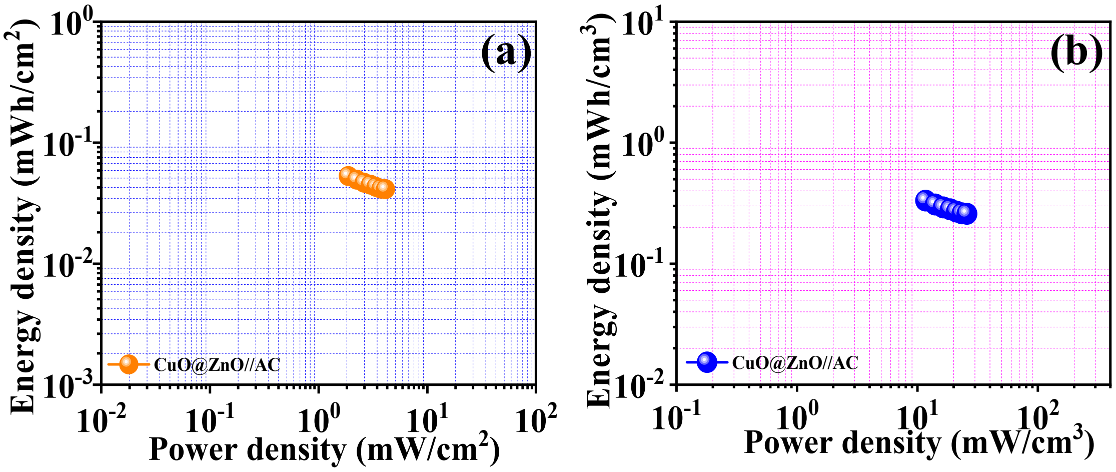

Figure S5: Ragone plot corresponding to C_a (a), and C_v (b) of CuO@ZnO//AC ASC.
